# The impact of reporting magnetic resonance imaging incidental findings in the Canadian alliance for healthy hearts and minds cohort

**DOI:** 10.1186/s12910-021-00706-3

**Published:** 2021-10-28

**Authors:** Judy M. Luu, Anand K. Sergeant, Sonia S. Anand, Dipika Desai, Karleen Schulze, Bartha M. Knoppers, Ma’n H. Zawati, Eric E. Smith, Alan R. Moody, Sandra E. Black, Eric Larose, Francois Marcotte, Erika Kleiderman, Jean-Claude Tardif, Douglas S. Lee, Matthias G. Friedrich, the CAHHM Study Investigators, S. Anand, M. Friedrich, J. Tu, P. Awadalla, T. Dummer, J. Vena, P. Broet, J. Hicks, J.-C. Tardif, K. Teo, B.-M. Knoppers, D. Desai, S. Nandakumar, M. Thomas, S. Zafar, K. Schulze, L. Dyal, A. Casanova, S. Bangdiwala, C. Ramasundarahettige, K. Ramakrishnana, Q. Ibrahim, D. Desai, H. Truchon, N. Tusevljak, K. McDonald, N. Noisel, J. Chu, J. Hicks, H. Whelan, S. Rangarajan, D. Busseuil, J. Leipsic, S. Lear, V. de Jong, M. Noseworthy, K. Teo, E. Ramezani, N. Konyer, P. Poirier, A.-S. Bourlaud, E. Larose, K. Bibeau, J. Leipsic, S. Lear, V. de Jong, E. Smith, R. Frayne, A. Charlton, R. Sekhon, A. Moody, V. Thayalasuthan, A. Kripalani, G. Leung, M. Noseworthy, S. Anand, R. de Souza, N. Konyer, S. Zafar, G. Paraga, L. Reid, A. Dick, F. Ahmad, D. Kelton, H. Shah, F. Marcotte, H. Poiffaut, M. Friedrich, J. Lebel, E. Larose, K. Bibeau, R. Miller, L. Parker, D. Thompson, J. Hicks, J.-C. Tardif, H. Poiffaut, J. Tu, K. Chan, A. Moody, V. Thayalasuthan, M. Friedrich, E. Smith, C. McCreary, S. E. Black, C. Scott, S. Batool, F. Gao, A. Moody, V. Thayalasuthan, E. Larose, K. Bibeau, F. Marcotte, F. Henriques, Jean Rouleau, Pierre Boyle, Caroline Wong, Eldon Smith, Bob Reid, Ian Janssen, Amy Subar, Rhian Touyz

**Affiliations:** 1 Arts and Science Program, McMaster University, 1280 Main Street West, Hamilton, ON L8S 4K1 Canada; 2 Population Health Research Institute, Hamilton Health Sciences, McMaster University, 237 Barton St East, Hamilton, ON L8L 2X2 Canada; 3 Department of Medicine, McMaster University, 1280 Main Street West, Hamilton, ON L8S 4K1 Canada; 4 Centre of Genomics and Policy, McGill University, 740 Dr Penfield Ave, Suite 5200, Montréal, QC H3A 0G1 Canada; 5 Department of Clinical Neurosciences, Hotchkiss Brain Institute, University of Calgary, Alberta, Canada; 6 Department of Medical Imaging, Sunnybrook Health Science Centre, University of Toronto, Toronto, ON Canada; 7 Department of Medicine (Neurology), Sunnybrook Health Sciences Centre, University of Toronto, Toronto, ON Canada; 8 Institut Universitaire de Cardiologie Et de Pneumologie de Québec - Université Laval, 2725 chemin Sainte-Foy, Québec, G1V 4G5 Canada; 9 School of Population and Public Health and Cancer Control Research, BC Cancer, University of British Columbia, 675 W 10th Avenue, Vancouver, BC V5Z 1L3 Canada; 10 Research Centre, Montreal Heart Institute, Université de Montréal, 5000 Belanger Street, Montreal, QC H1T 1C8 Canada; 11 Institute for Clinical Evaluative Sciences, Toronto, ON Canada; 12 Peter Munk Cardiac Centre, University Health Network, University of Toronto, Toronto, ON Canada; 13 Department of Medicine and Diagnostic Radiology, McGill University, 1001 Decarie Boulevard, Montreal, QC H4A 3J1 Canada

**Keywords:** Incidental findings, Magnetic resonance imaging, Quality of life, Ethics

## Abstract

**Background:**

In the Canadian Alliance for Healthy Hearts and Minds (CAHHM) cohort, participants underwent magnetic resonance imaging (MRI) of the brain, heart, and abdomen, that generated incidental findings (IFs). The approach to managing these unexpected results remain a complex issue. Our objectives were to describe the CAHHM policy for the management of IFs, to understand the impact of disclosing IFs to healthy research participants, and to reflect on the ethical obligations of researchers in future MRI studies.

**Methods:**

Between 2013 and 2019, 8252 participants (mean age 58 ± 9 years, 54% women) were recruited with a follow-up questionnaire administered to 909 participants (40% response rate) at 1-year. The CAHHM policy followed a restricted approach, whereby routine feedback on IFs was not provided. Only IFs of severe structural abnormalities were reported.

**Results:**

Severe structural abnormalities occurred in 8.3% (95% confidence interval 7.7–8.9%) of participants, with the highest proportions found in the brain (4.2%) and abdomen (3.1%). The majority of participants (97%) informed of an IF reported no change in quality of life, with 3% of participants reporting that the knowledge of an IF negatively impacted their quality of life. Furthermore, 50% reported increased stress in learning about an IF, and in 95%, the discovery of an IF did not adversely impact his/her life insurance policy. Most participants (90%) would enrol in the study again and perceived the MRI scan to be beneficial, regardless of whether they were informed of IFs. While the implications of a restricted approach to IF management was perceived to be mostly positive, a degree of diagnostic misconception was present amongst participants, indicating the importance of a more thorough consent process to support participant autonomy.

**Conclusion:**

The management of IFs from research MRI scans remain a challenging issue, as participants may experience stress and a reduced quality of life when IFs are disclosed. The restricted approach to IF management in CAHHM demonstrated a fair fulfillment of the overarching ethical principles of respect for autonomy, concern for wellbeing, and justice. The approach outlined in the CAHHM policy may serve as a framework for future research studies.

*Clinical trial registration* https://clinicaltrials.gov/ct2/show/NCT02220582.

**Supplementary Information:**

The online version contains supplementary material available at 10.1186/s12910-021-00706-3.

## Key Message

- The CAHHM policy for management of IFs followed a restricted approach, whereby routine feedback was not provided and only IFs of severe structural abnormalities were reported to participants who gave informed consent for disclosure.
- The overall satisfaction for participation in the study was high, as the majority would enrol in the study again and believed undergoing the MRI scan to be beneficial, regardless of whether they were informed of IFs.
- The disclosure of IFs mostly led to minimal or no impact on participants’ quality of life. However, for approximately half of participants, the disclosure of IFs was associated with increased stress, and for a small proportion, the disclosure of IFs did negatively impact their quality of life.
- The restricted approach to IF management demonstrated a fair fulfillment of the overarching ethical principles of respect for autonomy, concern for wellbeing, and justice.
- The presence of diagnostic misconception was found amongst the participants, indicating the importance of a more thorough consent process to support participant autonomy.
- The discovery of an IF did not result in any adverse consequences to life insurance policies for most participants.

## Background

With the advancement in computing power and new technologies over the last three decades, the use of medical imaging in research studies has rapidly accelerated [1]. However, inherent in the quantitative application and ability to capture a substantial number of data points, is the generation of incidental findings (IFs) that are beyond the intended aims of the research study, but may be of clinical significance to participants [2]. Due to the presence of IFs in MRI studies, it is widely accepted that investigators have an ethical obligation to account for potential IFs in the study protocol, discuss their possibility in the informed consent process, and plan how to address findings that may be of significance for research participants [3, 4]. However, the process by which individuals are notified of clinical IFs and the mechanism to disclose the information remains a complex and challenging issue [5–9].

Oren et al. in their recent publication, identified the challenge of incorporating IFs into clinical trial reporting and presented a call to action to better understand the implications of unanticipated imaging findings, as the use of medical imaging in research continues to increase [5]. The wide variety of approaches used in the communication and management of IFs in research indicates a need to better understand the ethical underpinnings of IF management, in order to approach these decisions with sufficient care [10–12].

In this section, the prevalence of IFs in MRI studies will be outlined, followed by an introduction to various ethical approaches that have been taken to address IFs in MRI research.

### Prevalence of incidental findings in MRI research studies

Incidental findings in MRI studies are common. In a recent meta-analysis of nearly 28,000 asymptomatic adults, the pooled prevalence of potentially serious IFs (defined as those that are likely to threaten life span, quality of life or major body functions) was 3.9% for brain and body MRI (1.4% brain, 1.3% thorax, and 1.9% abdomen) and further increased to 12.8% (1.7% brain, 3.0% thorax, and 4.5% abdomen) when including IFs of uncertain potential seriousness [13]. When compared to the UK Biobank imaging study, which evaluated total body MRI data from 1000 participants from the general population, potentially serious IFs were found in approximately 18% of the UK study participants when systematically reviewed by radiologists [14]. Furthermore, in the Multiethnic Study of Atherosclerosis (MESA) cohort, using coronary magnetic resonance angiography in 254 participants free of cardiovascular disease, IFs were detected in as high as 40% of the sample population. However, only 7% of participants had IFs of more clinical significance (grade 2 and above) [15]. The heterogeneity in the prevalence of IFs across various imaging studies is likely multifactorial and can be related to differences in participant baseline characteristics, imaging protocols, and definitions used for clinically significant IFs [13].

### Ethical approaches for the management of incidental findings

There is a general understanding that the obligation for researchers to report IFs to participants rests upon several principles, including the duty for researchers to respect participant autonomy, the duty to care for the well-being of participants, and the duty to treat participants fairly and equitably. Disclosing IFs demonstrates a respect for participant autonomy because it ensures that participants have access to personal, potentially relevant health information, which can enable them to make informed decisions about their own healthcare [16, 17]. Reporting IFs to research participants also supports the well-being of research participants, because discovery and disclosure of a life-threatening or life-changing condition could allow a participant to receive urgent, potentially life-saving treatment (Additional file 3: Supplementary Comment 1) [18]. The principle of justice is relevant to the management of IFs to ensure that researchers take a consistent approach to IF procedures and disclosures in order to treat participants fairly and equitably [19].

In light of these obligations, a debate has emerged in the literature regarding the ways in which IFs should be managed in imaging research. Some, such as Phillips et al., argue that researchers have an obligation to provide a full and universal review of MRI scans in research studies, and report all IFs to participants [17]. These authors contend that reviewing MRI scans for IFs is of low cost to researchers, participants have a strong desire to receive MRI information, and this information is usually of low harm to participants with serious potential benefits [17].

However, others have emphasized that a balance must be struck between the beneficial information attained by MRI scans and the potential drawbacks of IF disclosure, including misdiagnosis or overdiagnosis [20]. As Ells and Thombs describe, the discovery of IFs can be time-consuming, and the reporting of IFs can be costly for participants, in some cases with limited health benefit [18]. For example, IF disclosure has impacted participants’ ability to obtain insurance in some studies, which could be a costly scenario for participants being informed of an IF when the severity is unknown [17]. Another concern about the reporting of IFs is the anxiety caused by their disclosure [18]. Recent work from the Rotterdam study using detailed narratives of participants’ IF experiences revealed substantive impact on individual and family members’ well-being, related to familial stress and post-facto concerns about whether receiving the IF was worth it [16, 17]. Finally, automatically offering the return of IFs and in particular “all” potentially clinically significant IFs contributes to the diagnostic misconception in research, that is the failure to communicate and distinguish between research (generalizable knowledge) and medical care. These findings raise questions regarding the true harms experienced by participants in receiving IF feedback, and the responsibilities of researchers to try and mitigate this harm.

Due to these potential drawbacks of IF investigation and disclosure, many studies have chosen to take a restricted approach to the reporting of IFs—limiting which IFs are actively searched for (if at all) and which IFs are subsequently reported. Hegenscheid et al. chose to limit the reporting of IFs to merely those “with a high likelihood of relevant disease,” decided by an interdisciplinary advisory board [20]. Similarly, the UK biobank decided to limit their disclosure of incidental findings to “potentially serious” IFs which might pose a threat to the life span or quality of life of participants [21]. These restricted approaches attempt to balance the harms created by IF disclosure with the benefits gained by reporting IFs by limiting the number of IFs reported to only those of the most probable impact.

Within Canada, considerations for the management of IFs was catalyzed by the development of an ethical obligation to disclose “material” IFs to participants in research studies by the national Tri-Council policy statement back in (TCPS) 2010 [19]. This provision, however, did not provide practical details of how to navigate the complex issue for investigators. In 2018, the Tri-Council published substantial revisions to its policy statement (TCPS-2) on the issue of IFs and took a more directive approach [4]. Article 3.4 informs us that researchers have an *obligation* to disclose material IFs to participants who have agreed to receive them during the consent process. Material was defined as having analytical validity, clinical significance, and actionability [19].

As part of the commitment to uphold ethical practices in research, the Canadian Alliance for Healthy Hearts and Minds (CAHHM) created a policy for IFs discovered when volunteer participants underwent magnetic resonance imaging (MRI) scans. The CAHHM is a national, collaborative research effort between multiple cohort studies, including 13 MRI centres, which aimed to understand the association of socio-environmental and contextual influences with cardiovascular risk factors, subclinical vascular dysfunction, and other chronic disease outcomes [22]. Along with baseline data, which included collection of health information via questionnaires and physical measurements, participants underwent an MRI scan to evaluate cardiac, cerebral, and vascular function, allowing for potential, unexpected discoveries of structural abnormalities. CAHHM’s policy for incidental findings was formulated to ensure that IFs were managed in an efficient, consistent, and ethically-informed manner across all research sites.

While various aspects related to IFs have been studied, including associated lifestyle and socio-demographic risk factors [23], little is known about the impact of reported IFs on research participants’ quality of life, particularly in large, Canadian cohort studies such as the CAHHM [14]. Therefore, the objectives of this study were to first, describe the CAHHM policy for the management of IFs; second, to understand the impact of disclosing IFs to apparently healthy participants in the CAHHM cohort; and third, to reflect on the ethical obligations of researchers in future MRI studies.

## Methods

### Study population

The recruitment of participants followed the protocols published by the CAHHM investigators and included eligible adults between the ages of 30–69 years at the time of enrollment in their parent cohort [22], who had provided written informed consent for completion of study procedures and willingness to undergo a complete MRI scan that included brain, cardiac, carotid, and abdominal imaging. Participants were ineligible if they had contraindications to MRI, including claustrophobia, pregnancy, non-compatible pacemaker/defibrillator devices, and intraocular/intracranial metallic materials. The data included in this analysis refers only to the non-First Nations Alliance cohort. Research ethics approval was granted by the Hamilton Integrated Research Ethics Board and local REBs as appropriate, with consent obtained at each collaborating site prior to participating in the study, as per site-specific regulations.

### Framework for management of IFs

The approach taken to address material IFs in the CAHHM policy is built on a comparative analysis of international policies and upholds the ethical recommendations outlined by the national TCPS-2. These recommendations are grounded in the three core principles for ethical conduct in research; namely, Respect for Persons, Concern for Welfare, and Justice [19].

Respect for Persons refers to the obligations of researchers to respect the autonomy of research participants by ensuring that research participants have the ability to take part in research in an informed and free manner [19]. Research participants should have the ability to make their own decisions regarding their participation in research, and the research team has a responsibility to remain transparent and accountable for participants’ ability to do so. Concern for Welfare refers to researchers’ responsibility to consider the impact of research on participants’ mental, physical, and spiritual health, as well as their social and economic circumstances [19]. Over the course of a research project, this means that researchers are responsible for finding the best possible balance between beneficence—the benefits attainted from research participation—and non-maleficence—minimizing the harm caused by study participation. Finally, the principle of justice refers to researchers’ responsibility to treat participants fairly and equitably during research. In following this principle, participants should be regarded with equal concern, and the benefits and burdens as well as access to research must be distributed equitably amongst individuals [19].

### Informed consent process

One of the ways in which the obligation to respect participants’ autonomy can be fulfilled, according to the Tri-council policy, is by seeking free and informed consent from research participants [19]. The written informed consent process for the CAHHM was designed with the underlying principle of respect for participant autonomy; study information and IF protocols were clearly disclosed to participants, who were subsequently given the choice to decide whether or not to enroll in the MRI study and receive results about clinical IFs [24].

Firstly, participants were informed that the MRI scans and images obtained by experimental methods were for research purposes and therefore, not reviewed by a physician for diagnostic indications. Thus, routine feedback of individual results was not offered. The written consent form further outlined the possible risks and discomforts associated with the study, including the potential for the MRI scan to reveal a structural abnormality that may require medical treatment or preventive measures. In this circumstance, results were to be offered to the individual and their family physician (or another physician of their choice), if they so choose, as participants also had a right not to know. The right not to know was deemed a crucial aspect of the respect for participants’ autonomy, because the option to opt out of receiving IF information allowed participants to truly weigh the risks and rewards of IF disclosure, and make their own free decision regarding whether knowing or not was the best option for them. This ensured that, in accordance with the Tri-Council Policy statement, participants had the autonomy to decide which option would be most beneficial for their wellbeing. In cases where participants opted to receive information about a significant structural abnormality, these individuals were informed that their physician would be able to add the information to their medical records, which would then be potentially accessible by employers or insurers in the appropriate instances, once approved for release by the individual participant. The consent form specified in the event where an immediately life-threatening condition was demonstrated at any stage during the research study, participants were to be escorted to emergency care.

### The CAHHM policy for the management of incidental findings

Underlining the CAHHM policy was the desire to promote and safeguard the wellbeing of research participants [24]. As a large, multicentre research study, the ramifications of disclosing false positives and the potential negative impact on the psychological and financial wellbeing of participants were considered, especially if subsequent investigations did not reveal anything of actionable significance. As outlined in the literature, the reporting of IFs can lead to anxiety in research participants and their families, and can incur other social and financial costs, even when the IF is of unknown or benign significance [16, 18]. Furthermore, while not captured in our questionnaire, limited studies available with systematic follow-up data suggests that only 1 out of 5 individuals (20%) with a potentially serious IF eventually had a related significant, final clinical diagnosis [13]. Consequently, the CAHHM planning committee decided to employ a restricted approach, opting to not provide routine feedback and reported only information about severe structural abnormalities that were known to have impact on either longevity or quality of life, and where therapeutic options with proven benefits existed. In accordance with the principle of Concern for Welfare, this approach anticipated that participants could benefit from the IFs, and could treat or prevent dangerous underlying pathologies, while minimizing the undue harm that could be created by an over-reporting or misdiagnosing of IFs of minimal relevance. The CAHHM planning committee deemed this to be the most effective way of balancing benefits and costs from IFs, and the approach aligned with the Tri-Council policy’s obligation to report material incidental findings. This approach is also preferred by other large population-based studies, such as the UK Biobank [21] and allows for a reasonable balance of the ethical principles of autonomy—participants are informed of actionable structural abnormalities and can plan their healthcare accordingly—and non-maleficence—they are not faced with IFs of questionable significance which could incur social, financial, or psychological harm.

In order to ensure that participants’ welfare was adequately protected, however, an important aspect of the CAHHM policy was the consideration of time of reporting IFs. Promptness is a crucial aspect of the management of IFs because if a severe structural abnormality is found, individuals may have to act quickly to prevent or treat the finding. Due to the large-scale of the study, and the time required analyze and create reports for the discovered IFs, a reporting target of 3-months was created, which was deemed attainable and also sufficiently prompt to allow participants and their physician to address the finding in good time.

### Definition of incidental findings of severe structural abnormalities

Four imaging core labs separately evaluated the research MRI scans of specific anatomical regions, including the brain (University of Calgary and Sunnybrook Hospital), cardiac (Montreal Heart Institute), carotid (Sunnybrook Hospital), and abdomen (Institut universitaire de cardiologie et de pneumologie de Québec) using a standardized reading protocol. The standardized protocol ensured consistency and fairness in the IFs reported across participants, aligning with the Tri-Council’s principle of Justice in research. When an IF was discovered, the readers documented the type of finding, location, and extent of the abnormality. Only IFs of severe structural abnormalities were reported, which was defined as an abnormality that may potentially threaten lifespan, quality of life or major bodily functions, and for which there is a preventive or therapeutic strategy. These potential findings were also explicitly mentioned in the consent forms. Specifically, the following structural features were considered severe and reportable, if so desired: brain infarct excluding lacunes; myocardial infarction defined as high signal in late gadolinium enhanced images from extended MRI scan or a segmental wall thickening of < 10% (severe hypokinesis/akinesis) for at least 1 of the 16 standardized segments; aortic dilatation [thoracic > 50 mm (men) or > 45 mm (women) and abdominal > 45 mm (men) or 40 mm (women)]; valvular dysfunction (moderate or severe, with LV dilatation or dysfunction); and mass with positive criteria for malignancy or significant compression/infiltration of vital structures. The discretion was left to the MRI readers, who were all physicians of the specialties relevant to each structural abnormality, to determine whether a structural feature was severe. This meant that each MRI scan was read by four different physicians in four Core Labs, who were experts in MRI reading but not necessarily radiologists. The information was then reported to a central coordinating centre located at the Population Health Research Institute (PHRI), Hamilton Health Sciences and McMaster University where the information was linked to the individual participant’s clinical data, as was consented to by participants. This helped determine whether the finding reflected a previously diagnosed disease process or was a new finding, though the information was sent to the participant and family doctor regardless.

### Communication with participants and family physicians

By virtue of the Canadian universal health care system, family physicians are essentially gatekeepers and follow-up to all clinically relevant requests for bloodwork, imaging investigations, prescriptions, and referral to medical specialists. At this time, there exists very limited options to use a private route where an individual would pay for healthcare services in Canada. With this in mind, if a participant had provided written consent to receive information regarding the presence of any of the listed severe structural abnormalities, the discovery of an IF was reported back to the individual and their physician through a formal letter by the responsible site principle investigator (PI), ideally within a 3-month period after the MRI was conducted. The local investigator also telephoned the family physician to discuss the finding, its limitations given that it was a research scan, and possible additional testing. It was also the responsibility of the relevant site PI to document that the participant and their physician were sent all necessary information, so that they could determine if any further action was needed. The CAHHM policy mandated against additional research-funded scans due to funding limitations, but at the request from the individual’s physician, MRI scans were made available for consultation.

The option of receiving a report of an IF personally, without disclosure to a family physician was not offered to ensure that severe structural abnormalities could be handled by appropriate clinicians. However, in the case that a participant did not have a family physician or did not list one, the participant would then receive the information about a potential IF personally. In this scenario, the site investigator offered additional contact information for local physicians or a list of local walk-in clinics. This added measure aimed to ensure that all participants would have an opportunity to discuss their IF with a physician, and make informed, autonomous decisions about their healthcare. The discretion was left to the participant and their physician to determine whether additional investigations were needed, as they were informed that the scan was conducted for research purposes and had not been formally reviewed to obtain a clinical diagnosis.

### Follow-up questionnaire

Using an online survey, a short questionnaire of 9 open-text and multiple choice questions was administered 12 months after completion of the study to 350 participants recruited through the Montreal Heart Institute (MHI) Biobank from Montreal, Quebec, and 559 participants recruited into the CAHHM through the cohort of Chinese origin Canadians living in the greater Toronto area (GTA), Ontario region. The questionnaire asked about the general impact of participation in the CAHHM cohort, overall MRI experience, and the general course after receiving information about an IF. The questions for this survey can be found on Additional file 1: STable 1. Conducting the survey 12 months following study completion was deemed sufficient time to allow for return of potential IFs to participants and additional investigations, if deemed appropriate by the primary care team.

**Table 1:** Proportion of incidental findings discovered in the total sample population

	Scan of anatomical body part				Overall^c^
	Abdomen	Brain	Cardiac	Carotid	
Scans read for severe structural abnormalities	8196	8219	8188	8179	All scans read 8127
Scans not read or not available	62	39	70	79	
Total	8258	8258	8258	8258	Any scans read 8252
Total number of IFs discovered	257	346^b^	105	8	683
Mass	252	208	3	8	456
Myocardial infarction	4^a^		76		78
Aortic dilatation	1		7		8
Brain infarct		139			139
Valvular dysfunction			19		19

^a^Confirmed by extended scan

^b^One participant had both mass and infarct IF

^c^One count per participant

### Statistics analysis

Counts with proportions or means with standard deviations were provided for the follow-up questionnaire data and the overall study demographics, history of disease, and observed IFs. The Mantel Haenszel chi-square trend test was used to test the proportion of participants with an IF over decades of age. All analyses were completed using SAS version 9.4 (SAS Institute Inc, Cary, NC, USA).

## Results

Between 2014 and 2018, a total of 8,258 participants (mean age 58 ± 9 years, 54% women) were recruited with baseline demographics shown in Additional file 2: STable 2. Nearly all participants (99.7%) gave informed consent for disclosure of potential IFs (8235/8258). In total, 8.3% (95% CI 7.7–8.9%) (683/8252) participants were discovered to have at least one IF of severe structural abnormality, with the largest proportions found in the brain (4.2%; 346/8252), followed by the abdomen (3.1%; 257/8252), cardiac (1.3%; 105/8252), and carotid (0.1%; 8/8252) (Table 1). Having ≥ 2 IFs was rare (34/8252; 0.4%). The proportion of individuals increased with age, (*p* < 0.0001) (Table 2). Males had a higher proportion of any IF (9%) than females (7.6%), although this difference was accounted for with adjustment for age.

**Table 2:** Proportion of incidental findings by age

	30–39	40–49	50–59	60–69	70–79
N	136	1419	2987	2903	807
*Number of IFs*					
0	96.3% (131)	95.0% (1348)	93.2% (2783)	90.7% (2634)	83.4% (673)
1	3.7% (5)	4.9% (70)	6.6% (196)	8.8% (255)	15.2% (123)
2	0%	0.1% (1)	0.3%(8)	0.5%(14)	1.4%(11)

Mantel Haenszel Chi-square for trend, *p* < .0001

**Fig. 1:**
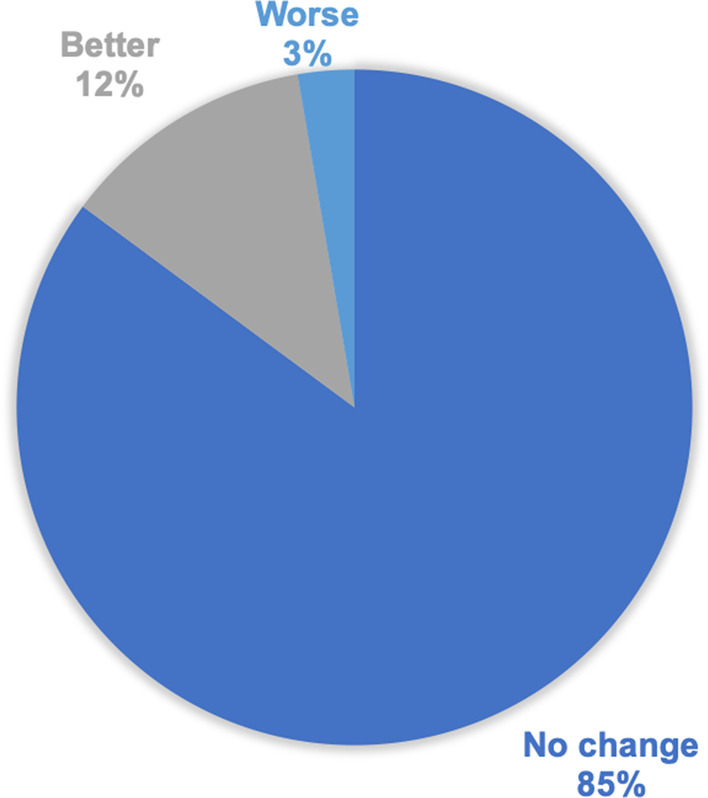
Reported change in quality of life in respondents with incidental findings of severe structural abnormalities (overall N = 74)

All participants from the two cohorts, MHI Biobank and Chinese cohort (n = 909), were invited to complete the follow-up questionnaire. The total response rate was 40% (357/909), and among the respondents, 21% (74/357) were informed about an IF and 79% (283/357) did not have IFs. Of those who were informed of a clinical IF, 85% (63/74) reported no change in quality of life since being notified of the finding, 12% (9/74) reported better quality of life, and 3% (2/74) reported his/her quality of life was worse (Fig. 1). Due to the clinical IF, 68% of participants (50/74) received additional scans and/or medical investigations. Of those 50 patients, 31 (62%) underwent a repeat MRI and 21 (42%) had another test performed, with 2 patients reported having both repeat MRI and other tests performed. Furthermore, because of the MRI findings, a small proportion of participants (8%; 6/74) had changes made to their medical treatment (2 patients had new medication/dose increased, 4 patients reported addition of new therapy/treatment) and 1 of the 6 individuals reported he/she experienced a side effect or complication because of the new therapy.

**Fig. 2:**
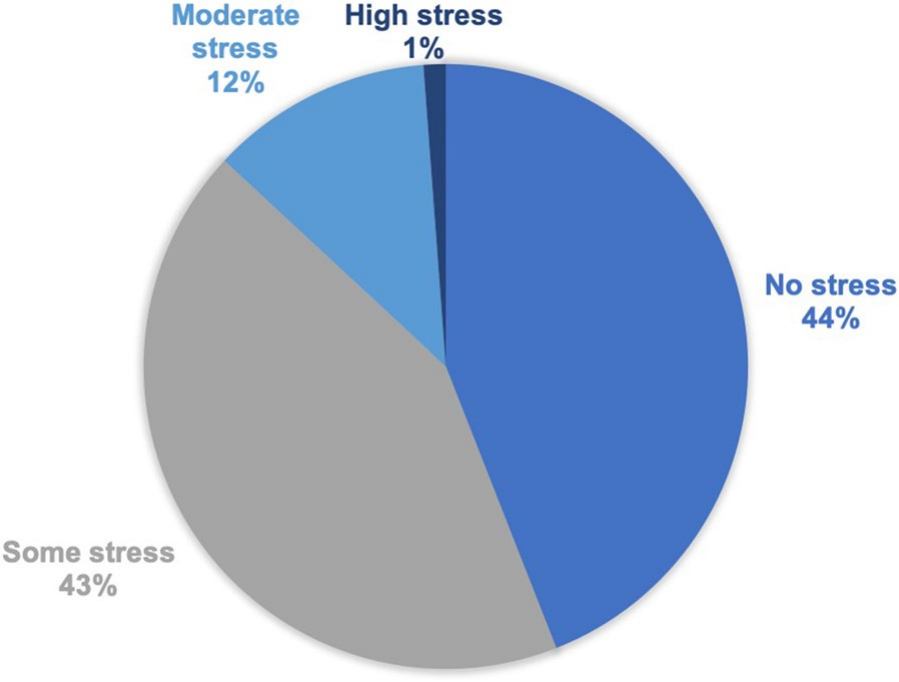
Degree of stress caused in respondents with incidental findings of severe structural abnormalities (overall N = 74)

In terms of stress, the report of an IF caused no stress in 50% (37/74) of respondents, some stress in 35% (26/74), moderate stress in 14% (10/74), and high stress in 1% (1/74) (Fig. 2). For most, but not all participants (95%; 70/74), the discovery of an IF did not result in any adverse consequences or changes to his/her life insurance policy. Of the 4 participants who reported the clinical IF resulted in change to their insurance policy, 2 individuals would neither enrol in the study again nor recommend the public get an MRI scan in general (the survey did not specify for research or clinical purposes). Three of these participants believed enrolment in the study was beneficial to their health.

Overall, when asked what the impact of participation in the study and undergoing the MRI scan was to their health, 23% (81/357) believed it was beneficial, 75% (347/357) answered neutral, and 3% believed participation in the study was harmful (10/357). Of those who were informed of an IF, 64% (47/74) believed participation in the study was beneficial to their health and 34% (25/74) answered neutral. In contrast, of those without IFs, 12% (34/281) believed participation in the study was beneficial to their health and 85% (239/281) answered neutral. Similar proportions (3%) of those who did and did not have IFs believed participation in the study and undergoing the MRI scan was harmful.

The length of time for reporting IFs to participants was optimized as the study progressed. The median time for reporting IFs was 242 days in the first year and improved to 43 days in the fifth and final year of the study. In the second year of the study, by becoming more familiar and efficient with the research protocol, as well as prioritizing timely reporting of IFs, the length of time for reporting IFs was reduced by more than half and reached a median of 100 days, which was close to the ideal period of 3 months. In the third year of the study, the median time for reporting IFs was 83 days, which was also within the 3-month reporting target.

### Participants’ free-text comments to the questionnaire

Specific examples of responses for participants who believed participation in the study was either beneficial or harmful are included in Table 3. Several important themes emerge from these responses regarding participants’ understandings of their MRI results, and the ways in which the study as a whole impacted their health. Firstly, numerous participants to whom IF abnormalities were disclosed expressed satisfaction from learning about their IFs and being able to manage their health accordingly. For example, one participant described that the discovery of a thyroid problem in the MRI scan allowed them to treat and monitor this problem, which was beneficial to their health and ability to manage their care. Other participants (not shown in Table 3) reported IFs which helped them treat and monitor abnormalities including cysts, a liver haemangioma, and a brain tumour. The responses regarding the success of the IF procedure in discovering abnormalities demonstrates that the restricted approach was successful at preventing harm and benefitting participants’ health, at least in some cases.

**Table 3:** Examples of participants’ responses to the question “Did you feel it beneficial, neutral or harmful to your health to take part in the Canadian Alliance for Healthy Hearts and Minds (CAHHM) and have the MRI scan?”

Response to question	Specific comments
Beneficial	Assurance that there are no major health problems at this level
Beneficial	At the psychological level, a personal security and a feeling of being able to help advance science
Beneficial	I became aware of some of my lifestyle habits
Beneficial	I feel reassured
Beneficial	I realized I needed to lose some weight… which is very positive…
Beneficial	Increased awareness of taking care of my mental and physical health
Beneficial	Reassuring that there were no major problems
Beneficial	MRI detected a thyroid problem that I can now treat and monitor
Beneficial	A mass has been detected, fortunately this is of no consequence (at least for the moment)
Beneficial	Possibility to have an MRI
Beneficial	Finding out nothing is wrong healthwise
Beneficial	It provides any medically significant finding so as to enable necessary follow-up
Beneficial	You didn't call me back, so everything was fine!
Harmful	I didn't like being in that machine too long on the inside.
Harmful	I found out I'm claustrophobic
Harmful	Stressful noise
Harmful	Headaches and dizziness more often
Harmful	How do you expect me to know? I haven't had any results from that magnetic resonance. I would have liked to have had more information on that test
Harmful	The next year I was diagnosed with breast cancer
Harmful	Harmful is not the right word, but there were consequences, as I was found to have a mass in the fourth ventricle of the brain which turned out to be an ependymoma and is under surveillance
Harmful	Because the MRI result didn't reveal that I have cancer in the left lung until I had a CT Scan a few days after the MRI

Another repeated theme from participants’ responses was the feeling of reassurance that participants experienced when they were not notified of IFs and major MRI abnormalities. Several participants felt assured that the lack of an IF report for them meant that they had no “major” health problems, which aligns with the goals of the IF procedure in reporting merely major abnormalities. However, it was clear that not all participants had an appreciation of the restricted approach taken in managing IFs. One participant believed that the lack of IFs in their MRI scans meant that “nothing was wrong healthwise,” while another felt the study was beneficial because it provided “any medically significant finding” to participants. These comments demonstrated that, at least for some participants, there was a misunderstanding regarding the restricted approach to IF reporting taken by the CAHHM study. Some participants seemed to believe that the lack of IF results meant that they were completely clear health-wise, which represents a phenomenon we termed ‘diagnostic misconception’, that is further analyzed in the Discussion.

Other reasons why participants felt the study to be beneficial included being able to advance science and partake in important research, as well as becoming more aware of their own lifestyle habits. This was an educational aspect of the study in this respect, as participants seemed to gain more knowledge regarding their lifestyle and physiological risks, and how they might go about living healthier lives. Generally, participants demonstrated a strong desire to learn more about their health and wellbeing through the imaging tests and the study questionnaires.

The participants who responded that participation in the study was harmful expressed several different concerns in the comments. A number of participants described negative experiences with the MRI procedure, including feeling claustrophobic and disliking the noise of the machine. Other concerns had to do more with the IF management process itself. For example, one participant expressed anxiety and agitation that they did not receive more information from the MRI scan. While the protocol and consent forms told participants that they would only receive the results if an abnormal finding was discovered, this suggests that some participants may have preferred communication whether or not a major IF was found.

Several of the participants’ concerns had to do with their IF diagnoses. One participant expressed concern after the IF reveled an ependymoma in their brain. The participant stated that this was under surveillance, and implied that this has caused some concern and anxiety. A second participant stated that they were diagnosed with breast cancer the next year, which suggests that they believed the MRI process missed an important abnormality which may have been actionable. A third participant expressed a similar concern about the MRI result not revealing a lung cancer which was discovered in a subsequent CT scan.. The participants’ concerns conceptualized through the free-text comments may represent instances of false-negative reports, in which material abnormalities were not reported to participants. The stories of these individuals suggest an assumption from participants that the study was responsible for reporting all abnormalities, which demonstrates a misunderstanding of the IF policy.

Overall, despite some of the concerns from participants, approximately 90% of the respondents (322/357) would participate in the study again and this did not differ between those who did or did not have IFs. Similarly, the majority (94%) responded affirmatively to the survey question which asked if they would recommend the general public get an MRI scan, regardless of whether they had an IF.

## Discussion

As outlined in the methods, the CAHHM IF management policy was predicated upon the Tri-Council’s three core principles for ethical conduct in research: Respect for Persons, Concern for Wellbeing, and Justice. As such, the aim of the framework was to optimize these responsibilities by ensuring participants’ autonomy was respected, the benefits of research participation were maximized while the harms were minimized, and participants were treated fairly and equitably. The CAHHM study results offer many important findings regarding how to optimize these principles in the management of IFs in future MRI studies.

A first important finding in our study, starting with the consent process, concerns the fact that 99.7 percent of participants agreed to be informed about IFs. This is an extremely high proportion of participants, especially considering the fact that there were risks associated with the disclosure of an IF. For example, participants were told that if they opted to receive IF information, their physician might add it to their medical records (once authorized by the participants), to which their employers or insurers could request access. It was also clarified that participants could opt out of receiving the information.

Assuming participation in the study had to do at least in part by a motivation to improve individuals’ own health, denying the disclosure of potential serious health problems may have seemed unwise for participants, particularly because imaging studies are expensive and difficult to access for healthy individuals. Judging from participants’ comments, it was clear that they felt a responsibility to themselves to look out for their health, and this was one of their main motivations for enrolling in the CAHHM study. Numerous participants described the benefits of participating in the study as having to do with the IF process, and learning “useful information” about their health. One participant even described the main benefit of the study as having the “possibility to have an MRI,” which indicates that the MRI scans and subsequent findings were important motivators for participants to join the study in the first place. This is consistent with other imaging studies such as the Rotterdam study, which reported that one of participants’ primary motivations for partaking in the study was to “[take] responsibility” for their health by receiving more information about their health status [16]. High participant consent to receiving IFs, then, could be an indicator of participants’ desire to learn about their health, and exercise their autonomy by obtaining imaging information about their bodies. In terms of the CAHHM policy as a whole, this affirms the fact that offering to report IFs supports participants’ autonomy when it comes to their healthcare, and participants have a large desire to receive this information.

Nonetheless, it was still surprising that only 3 in every 1000 participants opted not to receive the IF information. This huge discrepancy begs the question of whether participants had a true understanding of the benefits and risks of the IF process during consent. As Bomhof et al. describe, many participants of the Rotterdam study had not foreseen the possibility of receiving an IF and felt underprepared when they did receive a finding, even though they consented to IF disclosure and joined the study out of a desire to evaluate their health [16]. This suggests that many participants, even after reading and understanding the consent form, may not believe an IF is possible in their case, or do not sufficiently understand the prevalence and weight of these findings. This could lead many participants to consent to receive IFs without reflecting carefully on the benefits and harms of this information. The Rotterdam study described how participants felt that even with a more extensive discussion of IFs during the consent process, they would still feel underprepared for receiving the actual news. Despite this, however, it is possible that a more extensive discussion of the prevalence of IFs, and the benefits and risks of receiving IFs in the consent process, would allow some participants to reflect more carefully on their decision to receive IF information. This would support participants’ autonomy to make free and deeply informed decisions about their participation in research, including their right not to know about abnormalities, and would likely lower the extremely high percentage of participants who consented to receive IFs.

The results from our study also offered some important insights on the impact of the restricted approach to IF management. In our study of over 8000 participants, IFs of severe structural abnormalities found on MRI research scans occurred in 8.3% (95% CI 7.7–8.9%) of the sample population. For most participants, the disclosure of IFs led to minimal or no impact on the individual’s quality of life. However, for about half of these participants, the disclosure of IFs was associated with increased stress, and a small proportion of respondents reported that this knowledge did have a negative impact on their quality of life. The definition for quality of life and stress were determined by participants, who were asked how study participation impacted these aspects of their lives on an electronic questionnaire (Additional file 3: Supplementary Comment 2).

The discrepancy between individuals’ reports of stress due to IF reports, which was relatively high, and their reports on how IF reports impacted their quality of life, which was quite low, is a striking finding. Almost half of the participants reported some degree of stress from receiving feedback, and 15% experienced moderate to high degrees of stress, which is intuitively a contributing factor to an individual’s quality of life. One would expect that an individual experiencing health-related stress would have a worsened quality of life, yet this was not captured in the data. This might lend support to the finding from the Rotterdam study, which suggests that the real impact of IFs on individuals and their loved ones, revealed through open-interviews, might be more extensive than many studies report [16]. It is possible that while participants reported minimal impacts on their overall quality of life months after they received the IF feedback, this overarching report does not capture the true stresses and harms that individuals experienced between receiving their feedback and having a clinical diagnosis or treatment. While participants recalled experiencing stress—in some cases moderate or severe—it is conceivable that months later the questionnaire-based reporting of quality of life did not capture the short-term harms of receiving IF feedback. Another possibility to explain the discrepancy between quality of life and stress is that stress may not be considered an important aspect of quality of life for many people. Should this be the case, it challenges whether IF frameworks should consider participants’ experiences of stress at all in the management of IFs, if stress does not harm individuals’ quality of life.

The disappointment of participants from not receiving reports on certain abnormalities also relates to one of the most crucial findings of the CAHHM study—the prevalence of diagnostic misconception amongst participants. The concept of diagnostic misconception is an adaptation from the term ‘therapeutic misconception’, which refers to the situation whereby volunteers erroneously believe participation in a research study will provide knowledge about their health status, or incorrectly assume that the intent of research is therapeutic [25, 26]. When this is applied to imaging studies, diagnostic misconception concerns a failure to distinguish between research and clinical diagnosis. The occurrence of diagnostic misconception is problematic because it denotes a lack of understanding from participants about the risks and rewards of research participation, which is an essential part of participants’ autonomy when they decide to partake in a study. For example, in the CAHHM study, if participants believed that the primary goal of the study was to provide them with a health check so that they can prevent serious illness, they would be more likely to participate without considering the risks and limitations involved in the study. A diagnostic misconception can also harm participants, because it may create confusion with regards to their health status. In the CAHHM study, if participants believed that the MRIs were providing a thorough scan for primarily individual and diagnostic purposes, a negative scan could lead them to believe that they were completely clear health-wise, which may harm participants by creating a false sense of security. While participants may experience less anxiety in this case, they might also become less vigilant, and less likely to receive routine health check-ups, or be less likely to inquire into the relevance of any symptoms they were experiencing.

A sense of diagnostic misconception was conveyed most clearly through individuals’ responses to the questionnaire regarding the benefits and harms of participation. For example, participants who reported the MRI scan was beneficial to their health believed the study “provided useful information about [their] health”, and provided opportunities “to have an understanding of [their] current health status,” to “know more about [their] health,” or to “know [their] health is ok”. Consistent with previous studies, we suspect that participants may have perceived the absence of any reported IFs as an indicator of being healthy, which is a concerning finding since this was not necessarily the case and indicates a confusion between research and medical care, if not an inducement to participate [27]. If participants believed that the MRI results outlined in the consent form implied a thorough analysis of their precise health status, this may have led more participants to join the study without adequate reflection. It may have also resulted in participants who received a negative result to become overly relaxed about their health. It is crucial that participants have an adequate understanding of the benefits and risks of research participation, such that they can give their informed consent, and that they can manage their health in an informed manner. It is also important that participants do not perceive the report of IFs as providing a clinical-level scan of their health, but rather as notifying them if one of several specific abnormalities were found. While our consent forms clearly outlined the risks and benefits of study participation during the informed consent process, this suggests the need to have an even more thorough conversation with participants regarding the IF process. We recommend in future studies, researchers ensure that they discuss the disclosure of IFs very thoroughly during the consent process, and reiterate the distinction between research and clinical imaging before participants receive their MRI and potential IF results. Our study suggests that diagnostic misconception regarding the goals of imaging-based trials is very prevalent among study participants, which should be a key consideration in future MRI studies.

### Evaluation of the CAHHM framework

Overall, the results of the CAHHM framework offered some important lessons in the context of the Tri-Council Policy’s three principles of ethical research. The duty to respect participants’ autonomy began in the consent process, which outlined the MRI procedure, the types of abnormalities reported, and the potential implications of the abnormalities which individuals could consent to receive. This was important to ensure that participants understood the potential benefits and harms of the IF disclosure process before participating. Participants were also given the right not to know about severe structural abnormalities discovered during the MRI scans, which supported participants’ autonomy by ensuring that participants themselves decided which option would be most beneficial to themselves, rather than this decision being imposed on them by the research team.

Another important aspect of respecting autonomy was the decision to provide consenting individuals with information on material IFs that were deemed severe and actionable. This decision ensured that participants were informed of potentially clinically relevant information to help them make informed and autonomous decisions about their healthcare. In participants’ comments, they indicated that the disclosure of severe and actionable IFs allowed them to gain autonomy over their healthcare by highlighting issues that might require follow-up and monitoring. Participants felt that the findings enabled them to keep tabs on their health and choose more informed treatment options, which demonstrated the importance of disclosing significant IFs to respect participants’ autonomy and enable participants to manage their healthcare appropriately.

The CAHHM framework also raised some important considerations regarding how future MRI studies can further support participant autonomy. Participants’ comments in the follow-up questionnaire revealed a level of diagnostic misconception with their understanding of the limitations and aims of the IF process, and some individuals expressed disappointment that abnormalities discovered several months after the study were not reported from the MRI scan. These sentiments suggest that participants did not have a full understanding of the limitations of the MRI scans, which would in turn, limit their ability to make fully informed choices regarding their reception of IF information, and how they should proceed with their healthcare following disclosure (or lack thereof). We highlight this as an important area that will require extra attention for future MRI studies, and urge other researchers to consider additional effort to ensure that participants understand the true limitations of the consent process.

The next principle, Concern for Wellbeing—which includes the duties of beneficence and non-maleficence—was also central to the restricted approach to IF management. Firstly, as per the duty of easy rescue, as outlined by Koplin et al. [28], the restricted approach to IF management ensured that researchers informed participants of material IFs which were potentially life-saving or life-improving. In accordance with the duty of easy rescue, a reasonable attempt was made by the research team to both look for, and inform consenting participants of significant and actionable IFs which could be of great health benefit. The follow-up study found that 8% of participants received additional management for the IFs, indicating a significant number of participants who were able to avoid or treat health problems due to the MRI information. This demonstrated the success of the protocol in providing meaningful health benefits to the study population.

In terms of participants’ general reports of study benefits and harms, we found that the overall satisfaction for participation in the study was high, as the majority of individuals would enroll in the study again and believed undergoing the MRI scan to be beneficial, regardless of whether they were informed of IFs. This is consistent with previous literature, such as Gibson et al. in their study for the UK Biobank, who found over 95% of participants who received feedback about IFs were glad they were informed of the potentially serious findings and were glad to have taken part in the study [14, 27]. A very small percentage of participants (3%) in the CAHHM study reported that the study was harmful to their quality of life, which further indicates that the protocol was successful in minimizing harms experienced by participants.

With that said, almost half of participants reported that the disclosure of an IF caused them some stress, with 15% of participants reporting moderate to high stress. This indicates that participants who received IF reports experienced at least some harm, even if this was not reflected in their responses to the ‘quality of life’ question. This is a similar finding to other imaging studies, such as the population-based Study of Health in Pomerania (SHIP), where nearly 30% of participants reported moderate to severe psychological distress after being notified of an IF [25]. Additionally, in the UK Biobank, disclosure of IFs had an adverse impact on the emotional wellbeing for almost 20% of participants [14]. The relatively high likelihood of experiencing stress from IF reports indicates a benefit to the restricted approach to IF management, because this approach lowers the number of reports of minimal or unknown significance which are given to participants and could cause them unnecessary stress. The discovery of an IF did not result in any adverse consequences or changes to life insurance policies for most participants, but four participants out of the 74 with an IF did report that the IFs impacted their insurance. This is another harm experienced by participants due to the reporting of IFs, which can be minimized using a restricted approach.

A final aspect of harm which was indicated by participants’ comments was the presence of false-negative reports, or diagnoses of abnormalities after the study which were not reported in the IF feedback. While abnormal masses positive for malignancies were screened for in the IF detection phase, it is possible that small cancers were not deemed material enough to report, or the cancer might have developed following the conduction of the MRI, since it was not diagnosed until a year later. This is an important consideration because false-negative reports should be avoided in accordance with the duty of minimizing harm. Even if the abnormalities found by the two participants were not on the list of abnormalities examined in the study, they might have been found in a more extensive analysis of the scans and this remains a point of contention with restricted approaches to IF management. While the restricted approach can reduce harm by lowering the number of false-positive reports (as there are less reports overall), these benefits must be balanced with the harms of not reporting potentially significant abnormalities to some participants. We recommend that researchers in MRI studies pay particular attention to the prevalence of potentially-missed diagnoses in the future, so that researchers can be more informed when deciding which abnormalities are included in MRI analyses (Additional file 3: Supplementary Comment 3, Supplementary Comment 4).

For the principle of Justice, the restricted approach of CAHHM study was structured such that all participants received information about the same abnormalities, which ensured consistency and fairness across study participants. This is a benefit of the restricted approach, because it standardizes the analysis of IFs to minimize cases where some participants receive information about abnormalities that others do not. Finally, we also ensured that participants received IF information as quickly as possible, and we were able to decrease the time of reporting for all participants as the study progressed.

Overall, the management of IFs is a complex problem which requires a fine balance of many ethical considerations. In the CAHHM study, the restricted approach resulted in material health benefits for some participants, while minimizing the harm done by false-positives and the high levels of stress that IF reporting can create. Participants were very satisfied with their participation in the study, and an extremely small proportion of participants believed that participation in the study was harmful. The study demonstrated, however, that there are instances where researchers’ different ethical duties conflict in the management of IFs. For instance, while participants may prefer to know about all potential IFs, including IFs of minimal and unknown significance, researchers are also responsible for minimizing the harms that these reports can cause. This requires a careful balance of the principles of autonomy and non-maleficence. While the process to address IFs in clinical studies remains highly variable [29], the policy outlined by the CAHHM for management of severe structural abnormalities, and the lessons learned in the use of this policy, may serve as a framework for future research endeavors.

### Limitations

There are a few main limitations of this study. Firstly, only a small proportion of participants in the CAHHM cohort participants were offered the follow-up questionnaire due to logistical issues, and as such, there is a limited number of individuals with clinical IFs available for follow-up. Secondly, the questionnaire was designed to capture simple parameters related to the impact of the study, but was not extended to other stakeholders, including family physicians or family members. The questionnaire also did not capture the longer narratives of participants through more in-depth interviews, which has been shown to potentially reveal the costs related to IF disclosure to a greater extent [16]. The use of follow-up interviews, while time-consuming, will be an important next step to understand the true extent of the impact of IFs on research participants. Thirdly, the CAHHM study did not have full information on the outcome and final diagnoses of those with clinical IFs. Designing a follow-up to evaluate these outcomes would give us a better sense of the impact of IF reporting and the risk of false-positives. Lastly, with the current worldwide pandemic related to COVID-19 (Coronavirus disease 2019), the implications IFs will have for participants in ongoing studies and the complexity of how to safely navigate follow-up for all stakeholders will need to be considered.

## Conclusion

Policies to manage incidental findings obtained by research MRI scans remain a challenging issue, as participants may experience stress and a reduced quality of life when IFs are disclosed. Using a restricted, yet transparent approach to limit routine feedback and report only severe structural abnormalities to consenting participants respects both clinical and psychosocial factors. This analysis demonstrates that the restricted approach can strike a fair balance between the respect for participant autonomy, concern for their wellbeing, and the principle of justice in research. While we argue there is work to be done to understand the presence of diagnostic misconception in imaging studies, and the risks of false positive and negative reports, our policy extends the current literature and will help inform future research planning and studies of this kind.

## Supplementary Information

**Additional file 1. STable 1:** Online follow-up survey questions.

**Additional file 2. STable 2:** Baseline demographics of overall recruited participants and those with follow-up from the Chinese Canadians in the GTA and MHI Biobank cohort.

**Additional file 3. Supplementary Comments 1 to 4:** Additional background and discussion. 

## Data Availability

The datasets used and/or analysed during the current study are available from the corresponding author on reasonable request.

## Abbreviations

CAHHM: Canadian Alliance for Healthy Hearts and Minds
MRI: Magnetic resonance imaging
IF: Incidental finding
IFs: Incidental findings
REB: Research Ethics Board
PHRI: Population Health Research Institute
PI: Principle investigator
MHI: Montreal Heart Institute
GTA: Greater Toronto area
CI: Confidence interval
UK: United Kingdom
MESA: Multiethnic study of atherosclerosis
SHIP: Study of Health in Pomerania
COVID-19: Coronavirus disease 2019
