# Supplementary material for: The impact of reporting magnetic resonance imaging incidental findings in the Canadian alliance for healthy hearts and minds cohort
Source: BMC Med Ethics. 2021 Oct 28;22:145. doi: 10.1186/s12910-021-00706-3 (PMC8551943; doi:10.1186/s12910-021-00706-3)
Supplement: Supplementary file 1 — Additional file 1. STable 1: Online follow-up survey questions. [file 12910_2021_706_MOESM1_ESM.docx]

**Supplementary Table 1:** Online follow-up survey questions

| Did you feel it beneficial, neutral or harmful to your health to take part in the Canadian Alliance for Healthy Hearts and Minds (CAHHM) and have the MRI scan? | Beneficial: How______________  Neutral  Harmful: How_____________ |
| --- | --- |
| Having completed the CAHHM study, would you still have participated in the study? |  |
| Would you recommend that other members of the general public have an MRI scan? |  |
| During your participation at the CAHHM, you may have received feedback on some findings from your MRI scan. We are now trying to understand, how this affected you   1. Did you receive feedback on a potential finding from your MRI scan? | No: Thank you for your participation. SKIP remaining questions.  Yes: Please help us understand how this affected you, by answering the following questions from 2-6: |
| 1. Since we notified you and your doctor about the finding in the MRI scan, how has your quality of life changed? | Not changed  Better  Worse |
| 1. As a result of the MRI finding, did you have any additional scans or medical tests? | No. SKIP to question 4  Yes, please answer 3.1.1 and 3.2.1 below: |
| - 1. I had a repeat MRI      1. Did you experience any side effects or complications from this? | No  Yes |
| - 1. I had another test: (please specify): _________   3.2.1 Did you experience any side effects or complications from this? | No  Yes |
| 1. As a result of the MRI finding, did you receive a therapy or treatment or change of medication or treatment? | No, there was no new therapy or treatment or change in my current medication. SKIP to question 5.  Yes, please answer 4.1.1 and 4.1.2 below: |
| - - 1. I either received new medication, or my medication or the dose was changed.        1. Did you experience any side effects or complications from this? | No  Yes |
| 4.1.2 There was a new therapy/treatment: (please specify)  4.1.2.1. Did you experience any side effects or complications from this? | No  Yes |
| 1. Did the report of finding from the MRI cause you any stress: | No stress  Some stress  Moderate stress  High stress |
| 1. Did the finding have any other consequences such as a rejection or change of conditions of a life insurance contract | No  Yes, please specify: _____________ |
