# Supplementary material for: The impact of reporting magnetic resonance imaging incidental findings in the Canadian alliance for healthy hearts and minds cohort
Source: BMC Med Ethics. 2021 Oct 28;22:145. doi: 10.1186/s12910-021-00706-3 (PMC8551943; doi:10.1186/s12910-021-00706-3)
Supplement: Supplementary file 2 — Additional file 2. STable 2: Baseline demographics of overall recruited participants and those with follow-up from the Chinese Canadians in the GTA and MHI Biobank cohort. [file 12910_2021_706_MOESM2_ESM.docx]

**Supplementary Table 2:** Baseline demographics of overall recruited participants and of those with follow-up from the Chinese Canadians in the GTA and MHI Biobank cohort.

|  | **Overall** | **Chinese from GTA** | **MHI Biobank** |
| --- | --- | --- | --- |
| N | 8258 | 559 | 350 |
| Mean Age (SD), years | 57.9 (8.9) | 55.0 (9.1) | 57.2 (8.0) |
| Women | 54.1% (4466/8258) | 53.8% (301/559) | 46.9% (164/350) |
| Non-White Ethnicity | 18.3% (1512/8256) | 100.0% (559/559) | 0.3% (1/350) |
| Urban FSA | 97.5% (7936/8138) | 99.6% (557/559) | 92.6% (324/350) |
| Education: Any Post Secondary | 86.5% (6979/8064) | 91.6% (512/559) | 86.3% (302/350) |
| Married or Common Law | 75.5% (6083/8062) | 81.8% (457/559) | 77.1% (270/350) |
| Employed | 69.5% (5598/8057) | 65.7% (367/559) | 58.3% (204/350) |
| Retired | 23.3% (1881/8057) | 27.4% (153/559) | 39.1% (137/350) |
| History of Hypertension | 39.6% (3266/8246) | 34.2% (191/559) | 55.3% (193/349) |
| Taking Cholesterol-lowering statin medication | 19.8% (1629/8248) | 14.5% (81/559) | 36.6% (128/350) |
| History of Diabetes | 5.3% (437/8242) | 4.8% (27/559) | 7.8% (27/345) |
| History of Any CVD | 10.4% (861/8246) | 5.4% (30/559) | 33.5% (117/349) |
| History of Cancer | 6.8% (555/8131) | 4.7% (26/559) | 4.9% (17/350) |
| History of Major Depression | 9.5% (665/7014) | 5.7% (32/559) | 16.6% (58/350) |
| Family history of Cardiac disease | 33.7% (2780/8238) | 20.6% (115/559) | 46.3% (162/350) |
| Current smoking | 5.5% (450/8235) | 1.6% (9/559) | 4.6% (16/350) |
| Any CIF | 8.3% (683/8252) | 9.7% (54/559) | 10.9% (38/350) |
| Mass | 5.5% (456/8252) | 7.5% (42/559) | 3.7% (13/350) |
| MI | 0.9% (78/8252) | 0.5% (3/559) | 3.7% (13/350) |
| Aortic Dilatation | 0.1% (8/8252) | 0.0% (0/559) | 0.6% (2/350) |
| Valvular Dysfunction | 0.2% (19/8252) | 0.0% (0/559) | 0.9% (3/350) |
| Brain Infarction | 1.7% (139/8252) | 1.6% (9/559) | 2.0% (7/350) |
