# Supplementary material for: The impact of reporting magnetic resonance imaging incidental findings in the Canadian alliance for healthy hearts and minds cohort
Source: BMC Med Ethics. 2021 Oct 28;22:145. doi: 10.1186/s12910-021-00706-3 (PMC8551943; doi:10.1186/s12910-021-00706-3)
Supplement: Supplementary file 3 — Additional file 3. Supplementary Comments 1 to 4: Additional background and discussion. [file 12910_2021_706_MOESM3_ESM.docx]

**Supplementary Comment 1**

For Koplin et al., this responsibility is grounded in “the duty of easy rescue”—the moral obligation for a person to prevent significant harm to others, when it comes at a minimal cost to themselves.^1^ Koplin et al. argue that in accordance with the duty of easy rescue, researchers in imaging studies have a moral obligation to disclose IFs to participants and to actively *look* for IFs, so long as looking and reporting the IFs might prevent serious harm to the participant and comes at a small cost to the researcher.^1^

**Supplementary Comment 2**

The discrepancy between reports of stress and quality of life raises a crucial question in the management of IFs moving forward: which criteria should be used to determine the ‘harm’ done to participants in the process of reporting IF feedback? In this study, if stress is considered to be harm done to participants, the restricted approach used by CAHHM seems favourable, as almost half of the participants who received feedback experienced stress even with limitations on the types of IFs reported. If a larger number of IFs were reported, many more participants would have received IF reports, harming more participants due to the stress of these reports. This should be avoided due to the researcher’s responsibility of Concern for Wellbeing. However, if ‘quality of life’ is the indicator used to measure harm, the restricted approach used by CAHHM might do more harm than good to participants, because an under-reporting of IFs would be occurring due to an unsubstantiated fear of harming participants with IF feedback. In this case, a more broadened approach to IF management might best reduce the harms experienced by participants by ensuring that all possible material IFs are investigated and false negatives are best minimized. Measuring ‘harm’, albeit complex, is an important aspect of determining which approach to IF management best fulfills researchers’ duty to protect the well-being of research participants. The CAHHM study suggests that a deeper examination into this issue will be required moving forward to determine which frameworks for IF management are truly in participants’ best-interest.

**Supplementary Comment 3**

Another crucial factor to minimize harm in CAHHM’s restricted approach was managing the risk of false positives and false negatives in the study. One of the advantages of limiting the list of IFs reported to simply five pre-designated abnormalities was the avoidance of false-positive reports, including IFs of minimal significance. Given the high proportion of participants who experienced stress due to the IF reports, attempting to minimize false-reports was crucial in limiting the undue harms experienced by the participants and their loved ones. While it is impossible to know how many IFs of unsure or minimal significance were avoided in this study, the restricted approach was created with an understanding that a low percentage of individuals with potentially serious IFs have historically been found to have related clinical diagnoses.^2, 3^ In the CAHHM study, 8.3% of participants had an IF of severe abnormality, and the majority (68%) of those from the follow-up who received a clinical IF report received additional investigations. However, of these patients, only a small percentage (8%) reported changes to their medical treatment. These data suggest that while many of the participants who received IF reports followed them up with their physician, the MRI findings only led to significant medical changes for a small number of patients which indicates at least some level of false-positive reports. While this is surprising given the restrictions of IF reporting to only severe abnormalities, it also implies that with a more expansive IF framework, many more individuals would have likely received false-positive IF reports, which could be unnecessarily harmful to them. The fact that the restricted approach led to less than 10% of participants receiving IFs, which is much lower than similar studies such as the UK Biobank or the MESA cohort, suggests some success in limiting the harms of false-positive reports. This is supported by the fact that those who received IF reports were no more likely to believe participation in the study was harmful, and none of the participants’ comments suggested that their reason for reporting harm was due to a false-positive report. Overall, our study suggests that restricted approaches to IF management can still yield false-positive IFs, but the restricted approach limits these reports by decreasing the number of participants who receive IFs as a whole.

**Supplementary Comment 4**

Of course, attempting to limit false-positives by restricting the reporting of IFs to material and actionable abnormalities presents the risk of increasing the number of false-negative reports. While the raw number of false-negative reports was not captured in this study, two individuals from the follow-up questionnaire (0.56%) reported that they found the study was harmful to their health because they were later diagnosed with cancers that were not discovered in the MRI scan. This suggests that a small number of participants may have had abnormalities which were potentially not captured and reported from the MRI scans, which could potentially produce harmful consequences to participants if these abnormalities were left untreated. It was an extremely low percentage of individuals who reported these unreported abnormalities (i.e. possible false-negatives), but it still highlights the importance of managing the number of false-positives with the number of false-negatives in IF management. While a less restricted approach would better limit the number of false-negative reports, and ensure that potentially significant abnormalities are not missed for participants, these approaches would also be more likely to report a larger number of false-positives, which would place unnecessary stress on participants. An important question for future MRI studies concerns the way in which the harms of over-reporting and under-reporting are balanced. In the CAHHM study, it was found that while the restricted approach may have led to an extremely low number of false-negative reports, it was successful in providing some actionable findings to individuals, and the restricted framework likely limited a large number of false-positives. The very small percentage of individuals who believed that participating in the study and receiving an MRI was harmful (3%) suggests that the approach did a fair job managing the harms of reporting IFs at either extreme. Our results indicate a need to more closely examine the proportion of reported IFs that are falsely-positive and falsely-negative, and how to mitigate these occurrences, to continue to manage IFs in a way that best supports participant wellbeing.

**References**

1. Koplin JJ, Turner MR, Savulescu J. The Duty to Look for Incidental Findings in Imaging Research. *Ethics Hum Res*. Mar 2020;42(2):2-12. doi:10.1002/eahr.500043

2. Gibson LM, Paul L, Chappell FM, et al. Potentially serious incidental findings on brain and body magnetic resonance imaging of apparently asymptomatic adults: systematic review and meta-analysis. *Bmj*. Nov 22 2018;363:k4577. doi:10.1136/bmj.k4577

3. Vogel-Claussen J, Li D, Carr J, et al. Extracoronary abnormalities on coronary magnetic resonance angiography in the multiethnic study of atherosclerosis study: frequency and clinical significance. *J Comput Assist Tomogr*. Sep-Oct 2009;33(5):752-4. doi:10.1097/RCT.0b013e318196bf2e
